# Analysis of age-dependent trends in Ov16 IgG4 seroprevalence to onchocerciasis

**DOI:** 10.1186/s13071-016-1623-1

**Published:** 2016-06-13

**Authors:** Allison Golden, Dunia Faulx, Michael Kalnoky, Eric Stevens, Lindsay Yokobe, Roger Peck, Potochoziou Karabou, Méba Banla, Ramakrishna Rao, Kangi Adade, Richard G. Gantin, Kossi Komlan, Peter T. Soboslay, Tala de los Santos, Gonzalo J. Domingo

**Affiliations:** Diagnostics Global Program, PATH, Seattle, WA USA; National Onchocerciasis Control Programme, Kara, Togo; Onchocerciasis Reference Laboratory, National Institute of Hygiene, Sokodé, Togo; Institute of Tropical Medicine, University Clinics of Tübingen, Tübingen, Germany; Washington University, St. Louis, MO USA

**Keywords:** Onchocerciasis, Diagnostics, Neglected tropical diseases, Seroconversion, IgG4

## Abstract

**Background:**

Diagnostics provide a means to measure progress toward disease elimination. Many countries in Africa are approaching elimination of onchocerciasis after successful implementation of mass drug administration programs as well as vector control. An understanding of how markers for infection such as skin snip microfilaria and *Onchocerca volvulus*-specific seroconversion perform in near-elimination settings informs how to best use these markers.

**Methods:**

All-age participants from 35 villages in Togo were surveyed in 2013 and 2014 for skin snip *Onchocerca volvulus* microfilaria and IgG4 antibody response by enzyme-linked immunosorbent assay (ELISA) to the *Onchocerca volvulus*-specific antigen Ov16. A Gaussian mixture model applying the expectation-maximization (EM) algorithm was used to determine seropositivity from Ov16 ELISA data. For a subset of participants (*n* = 434), polymerase chain reaction (PCR) was performed on the skin snips taken during surveillance.

**Results:**

Within the 2,005 participants for which there was Ov16 ELISA data, *O. volvulus* microfilaremia prevalence and Ov16 seroprevalence were, 2.5 and 19.7 %, respectively, in the total population, and 1.6 and 3.6 % in children under 11. In the subset of 434 specimens for which ELISA, PCR, and microscopy data were generated, it was found that in children under 11 years of age, the anti-Ov16 IgG4 antibody response demonstrate a sensitivity and specificity of 80 and 97 %, respectively, against active infections as determined by combined PCR and microscopy on skin snips. Further analysis was performed in 34 of the 35 villages surveyed. These villages were stratified by all-age seroprevalence into three clusters: < 15 %; 15–20 %; and > 20 %. Age-dependence of seroprevalence for each cluster was best reflected by a two-phase force-of-infection (FOI) catalytic model. In all clusters, the lower of the two phases of FOI was associated with a younger age group, as reflected by the seroconversion rates for each phase. The age at which transition from lower to higher seroconversion, between the two phases of FOI, was found to be highest (older) for the cluster of villages with < 15 % seroprevalence and lowest (younger) for the cluster with the highest all-age seroprevalence.

**Conclusions:**

The anti-Ov16 IgG4 antibody response is an accurate marker for active infection in children under 11 years of age in this population. Applying Ov16 surveillance to a broader age range provides additional valuable information for understanding progression toward elimination and can inform where targeted augmented interventions may be needed. Clustering of villages by all-age sero-surveillance allowed application of a biphasic FOI model to differentiate seroconversion rates for different age groups within the village cluster categories.

**Electronic supplementary material:**

The online version of this article (doi:10.1186/s13071-016-1623-1) contains supplementary material, which is available to authorized users.

## Background

Onchocerciasis or “river blindness” is a neglected tropical disease caused by infection with the parasitic nematode *Onchocerca volvulus* (Ov). The disease affects approximately 37 million people in Africa and the Americas; more than 500,000 people are visually impaired and 250,000 people are blinded by the disease, with the majority of the disease burden in Africa [1–5]. The donation of the anti-parasitic medicine ivermectin, by Merck (Kenilworth, New Jersey, USA), has enabled the development of large mass drug administration (MDA) programs to reduce the burden of the disease. MDA combined with vector control has been successful in reducing transmission to elimination in the Americas [6–8]. Similar trends have been observed in foci in Africa resulting from large-scale implementation of vector control and MDA by the Onchocerciasis Control Program (OCP) and the African Program for Onchocerciasis Control (APOC) [9–13].

Monitoring progression to elimination requires measuring parasite presence (or confirming absence) in the vector and in the host as a means to confirm reduction in parasite transmission to below sustainable levels. In 2001 the Onchocerciasis Elimination Program for the Americas published guidelines for certification of elimination [14]. In these guidelines, the entomological threshold for declaring interruption of transmission is an upper bound of the 95 % confidence interval for prevalence of vectors carrying Ov infective-stage larvae L3 of less than 1/2,000 per endemic community, and the human threshold is a five-year cumulative incidence rate of infection of less than one case per 1,000 susceptible children, which can be demonstrated by skin snip microscopy, polymerase chain reaction, or immunological assays. Several markers for infection have been used for mapping, measuring disease burden, and certifying elimination, but many questions remain how to best use these markers in settings approaching elimination [15–17]. Skin snips are relatively insensitive when microfilarial (MF) skin densities are low. Polymerase chain reaction (PCR) of the skin snips may provide greater sensitivity but still require sampling skin snips [17–20]. Screening tests using skin snip samples is challenging to implement at large scale due to the relatively labor-intensive nature of the process, the invasiveness, and as local disease burden decreases, a lowered acceptability from the community to be subjected to this process. A transdermal patch that delivers diethylcarbamazine as a local microfilaricide that induces a local skin reaction (a Mazzotti reaction) also can be used as a marker for infection [13, 21, 22].

Serological markers are widely used to determine exposure to a disease. The most developed and advanced serological marker for exposure to onchocerciasis is IgG4 response to the *O. volvulus* marker Ov16 antigen that is expressed by the larval stages (L3 and L4) of the parasite [23]. In the Americas, the immunological assay measuring anti-Ov16 IgG4 seroconversion by enzyme-linked immunosorbent assay (ELISA) in children has been used widely to demonstrate interruption of transmission [7, 24, 25]. In Africa, increasingly the anti-Ov16 marker is used to confirm interruption of transmission in foci that received extensive rounds of MDA [6, 9, 12, 26]. The anti-Ov16 IgG4 assay is transferable to the nitrocellulose rapid diagnostic test (RDT) platform [27–29] and is now commercially available (Alere SD BIOLINE Onchocerciasis IgG4 Rapid Test, Suwon, Republic of Korea). The availability of the assay on a point-of-care RDT platform and the development of reagents to facilitate in-country training and support quality assurance greatly enhance the operational utility of the assay for surveillance activities post-MDA as well as possibly for monitoring progression toward elimination and mapping purposes [30].

In West Africa many countries, including Togo, under the Onchocerciasis Control Program (OCP) launched in 1974, successfully controlled onchocerciasis, initially through intensive vector control and subsequently through MDA with ivermectin [31–34]. In some countries, such as Mali and Senegal, elimination appears feasible [35, 36]. Foci in Togo that have received over 20 rounds of ivermectin appear also to be achieving elimination. The ability to identify regions that have been less successful at blocking transmission, despite intensive vector control as well as the multiple rounds of ivermectin, will be important to informing where and how to focus interventions. Understanding how the currently available markers for infection can be used in this context is important. Despite the broad utilization of the Ov16 serological marker in the Americas and even in Africa to confirm no ongoing transmission through serological surveys with children, there are very little cross-sectional, all-age anti-Ov16 data. All-age data allow the assessment of Ov16 endemicity over time by application of force of infection (FOI) analysis. This analysis is a simple reversible catalytic conversion model that implements an estimate for rate of infection, seroconversion, and seroreversion [37]. This study describes Ov16 serological data collected in Togo in the years 2013 and 2014 across 34 communities and spanning all ages above five years of age. Serological data are compared to microfilaria detection by microscopy and, for a subset of specimens, PCR. Serological data were analyzed using FOI models age- and village prevalence-dependent parameters of seroprevalence.

## Methods

### Surveillance studies in Togo

Two studies were performed in Togo to determine Ov16 seroprevalence by ELISA from dried blood spots (DBS) and to evaluate early prototype versions of an Ov16 RDT. Both studies were performed during routine onchocerciasis surveillance during the early rainy season just prior to annual MDA: June 4 to July 1, 2013 and May 11 to June 16, 2014. The studies were performed by staff members of the Togo National Program for Onchocerciasis (PNLO) and the Laboratory for Onchocerciasis Research (LRO). For each study, the staff received a two-day training course for performing study procedures including appropriate consent, data management, and utilizing the dried-down recombinant positive control [30]. In addition, a research team member was present for the first several hundred study participants to monitor test use and to provide ongoing support. Briefly, in the 2013 study, study participants were recruited in 15 villages over a period of 27 days, and in the 2014 study, in 20 villages over a period of 35 days.

### Collection of dried blood spots for Ov16 ELISA

Following consent, study participants were given a finger prick and resulting blood was collected on Whatman 903 Protein Saver Cards (GE Healthcare, Pittsburgh, PA, USA). The cards were stored in resealable mylar pouches (ten cards per pouch) containing two-unit clay desiccant packets (Desiccare, Reno, NV, USA) and a humidity indicator card. Dried blood spots (DBS) were returned to the LRO within 14 days of collection and stored at 4 °C until handled for ELISA.

Microfilaria status of each study participant was determined by microscopy of two corneoscleral punches (Holth- or Walser-type) of the skin collected from the right and left iliac crest and was conducted by the National Onchocerciasis Control Programme of Togo. Skin biopsies were immediately placed on flat-bottom glass slides and incubated in 0.1 ml physiological saline solution; after 30 min, each biopsy was examined microscopically and the emerged microfilariae of *O. volvulus* counted. After microscopy, the examined skin biopsies and saline were transferred individually into a single round-bottom well of a 96-well plate, submerged in saline solution, and after overnight incubation at room temperature; each biopsy was re-examined as described above. Final counts of *O. volvulus* MF/mg were recorded. Following microscopy, skin punches were returned to the LRO in Sokode, transferred with saline to 1.5-ml microcentrifuge tubes, sealed, and stored at -20 °C in the saline buffer until later use in PCR.

### Ov16-positive control DBS for ELISA

A solution of 250 ng/ml anti-Ov16 positive control antibody in fetal bovine serum (FBS) was prepared by dilution of a stock solution of 1 mg/ml anti-Ov16 recombinant IgG4 clone AbD19432_hIgG4 in 1X phosphate buffered saline (PBS), pH 7.4 (Bio-Rad AbD Serotec, Puchheim, Germany) into FBS (Invitrogen, Grand Island, NY, USA). Blood samples containing the anti-Ov16 positive control antibody were made by mixing the solution of 250 ng/ml thoroughly at a 1:1 dilution with packed, washed, red blood cells, and 75 μl per circle marking of the contrived whole blood sample was spotted on Whatman 903 Protein Saver Cards (GE Healthcare). These cards were dried overnight in ambient laboratory conditions and then stored at -20 °C with approximately ten cards per sealed mylar pouch containing two-unit clay desiccant packets (Desiccare, Reno, NV, USA).

### Horseradish-peroxidase (HRP)-developed ELISA for Ov16 IgG4 in DBS

DBS cards were punched using a 6-mm punch in a blood-saturated region and punches were arrayed into a round-bottom 96-well microtiter plate. Remaining card material was returned to the desiccant pouch and stored at 4 °C. One punch of positive control DBS containing 250 ng/ml Ov16 positive control was included for each plate eluted. Each punch was eluted overnight at 4 °C in 200 μl of PBS + 0.05 % Tween-20 (PBST) + 2 % (w/v) dry milk powder (Mix’n Drink, Saco, Middleton, WI, USA). The solution was mixed gently before use in ELISA. For Ov16 ELISA, 100 μl of 5 μg/ml Ov16 antigen diluted in PBS, pH 7.4 (Sigma Chemical Company, St. Louis, MO, USA) was added to plate wells, Immulon 2HB (Fisher Scientific, Pittsburgh, PA, USA) [30]. For the glutathione-S-transferase (GST) ELISA, 100 μl of 2 μg/ml GST protein (Thermo Fisher, Waltham, MA, USA) was added to plate wells. Plates were then covered and stored overnight at 4 °C. The following morning, plates were blocked with PBST + 5 % FBS (FBS - Invitrogen) at 37 °C. Plates were washed three times with PBST (Sigma Chemical Company, St. Louis, MO, USA) and 50 μl of DBS sample eluate was added without dilution to the plate wells. Each eluted punch supplied two replicate wells in the Ov16 ELISA and one well in the GST ELISA. Samples were incubated at 37 °C for one hour, then washed three times with PBST. A 1:5,000 dilution of an anti-human IgG4 (6025 clone Hybridoma Reagent Labs, Baltimore, MD, USA) was added at 50 μl per well. Plates were incubated at 37 °C for one hour, and washed four times with PBST. A 1:10,000 dilution of an HRP-conjugated goat anti-mouse antibody (Jackson Immuno Research Labs, West Grove, PA, USA) was added at 50 μl per well. Plates were incubated at 37 °C for one hour and then washed four times with PBST. One hundred μl of TMB (Sigma) solution was added to each well. Plates were incubated at room temperature for 15 min and then the reaction was stopped by adding 50 μl per well of 1 N HCl (Thermo Fisher). Plates were read at 450 nm.

### Polymerase chain reaction (PCR) of skin snips

For DNA extraction, the skin biopsies from field surveillance were removed from -20 °C storage and transferred to microcentrifuge tubes. Biopsies were processed using the Qiagen DNA Investigator kit (Qiagen, Hilden, Germany) according to the Tissues protocol, digesting the skin with proteinase K overnight at 56 °C. Three elutions, of 60 μl each, were performed. The eluted DNA concentration for each sample was determined by absorbance at 260 nm and DNA was stored at -20 °C before PCR analysis. Extracted skin biopsy DNA concentrations ranged from 4 ng/μl to 166 ng/μl. Real-time PCR primers and probe used were the following: OvFWD 5'-TGT GGA AAT TCA CCT AAA TAT G-3', OvREV 5'-AAT AAC TGA TGA CCT ATG ACC-3', OvProbe 5'-6FAM-TAG GAC CCA ATT CGA ATG TAT GTA CCC-MGBNFQ-3' (minor groove binding TaqMan® Probe #5208995 P/N 4316033, Applied Biosystems, Thermo Fisher) (R. Rao, unpublished data). Primers and TaqMan probe sequences were designed to amplify a fragment of *O. volvulus* repeat DNA (0–150 bp, GenBank acc. number: J04659.1) using Beacon Designer^TM^ from PREMIER Biosoft (Palo Alto, CA, USA). Taqman Universal PCR Master Mix (Applied Biosystems, P/N 4304437) and nuclease-free water were used with all reactions with the following concentrations and volumes: 2.5 μl of 20 μM OvFWD, 2.5 μl of 20 μM OvREV primer, 1.5 μl of 9.2 μM OvProbe, 27.5 μl of 2× Master Mix, 50 ng of template DNA from extracted skin biopsies, or 1 μl Ov150 positive control plasmid at 10 ng/μl (GenBank acc. number J04659.1), or 1 ng of genomic DNA isolated from adult *O. volvulus*, and nuclease-free water was added up to a final volume of 55 μl. Reactions (2 × 25 μl per well) were run with the following cycling conditions: 50 °C for 2 min, 95 °C for 10 min, (95 °C for 15 s, 49 °C for 30 s, 60 °C for 2 min) × 40 cycles.

Skin snips from participants in the 2013 study were run in single wells and analyzed using the Applied Biosystems 7300 Real Time PCR System (96-well format), SDS version 1.4 software. Skin snips from participants in the 2014 study were run in duplicate and analyzed using the Corbett Rotor Gene RG-300, version 6 software. Skin snip-derived DNA samples producing a Ct value of less than 29, in duplicate, were considered to be positive for presence of *O. volvulus* DNA.

### Statistical data analysis and modelling

Ov16 ELISA replicate optical density (OD) values were subjected to quality control criteria for plate control quality, sample replicate quality, and relative ELISA reactivity with the GST protein. Quality control steps were performed separately for each year’s dataset. Quality control requirements were as follows in order for specimen results to be included in the final dataset: all plates’ positive control OD values were within 1.5× of interquartile range (IQR) of the total dataset’s respective controls. If a plate was excluded, samples within that plate were also excluded and when possible, the plate was repeated. For individual specimens, the relative difference (replicate difference divided by mean of the replicates) of replicate OD values had to be less than 1.5× IQR of all specimen relative differences. The companion GST ELISA results were less than 1.5× IQR of all GST ELISA OD values. Those with significantly high GST values (> 1.5× IQR) were classified as those for which cross reactivity with GST tag on the recombinant Ov16 antigen could not be excluded and they were not included in the final analysis. For all specimens which had acceptable Ov16 ELISA data, the mean OD of the Ov16 ELISA replicates was normalized to the respective plate positive control OD and the 2013 and 2014 datasets were then combined. A univariate expectation maximization (EM) algorithm was applied to the normalized Ov16 ELISA dataset OD measurements characterizing the two subpopulations in the data as either Ov16 positive or negative. The EM algorithm was set to model the normalized distribution of OD measurements with two Gaussian curves, producing mixing probabilities of 0.79 and 0.21, means of 0.10 and 1.01, and variances of 0.002 and 0.825 for negative and positive distributions, respectively.

Force of infection analysis was applied by fitting the seroprevalence as determined by Ov16 ELISA to a reversible catalytic model [37, 38], as stratified by age and village prevalence ranges. Errors for estimates of prevalence by age were assumed to be binomially distributed. Optimal fitting to empirical data occurs of the FOI model and parameters lambda (seroconversion) and rho (seroreversion) were calculated using maximum likelihood techniques [39].

## Results

### Study demographics and microfilaremia

Surveillance activities were performed in 2013 and 2014 in Togo in 35 onchocerciasis-endemic villages, just prior to annual MDA. Thus the study participants had all received their previous dose of ivermectin over 11 months ago. Longitudinal MDA and vector control data were not available for all villages, but out of 15 villages for which the data were available, five villages had received MDA since 1992 and ten since 1997. All of them had received intensive vector-control interventions. During the surveillance activities, skin snip microscopy was performed on the same day and repeating at 24 h. DBS were collected for Ov16 ELISA. The same skin snips used for microscopy were preserved for PCR. A total of 2,927 study participants were recruited and provided skin snip and DBS samples (1,456 participants in 2013 and 1,471 in 2014). Overall 56 % of the study participants were female. The summary of the demographics and the number of MF-positive cases by age group is shown in Table 1.

**Table 1:** Study demographics broken out by age, total recruitment and number of participants included in the seroprevalence data analysis

Age range	Participants recruited		Participants with Ov16 IgG4 ELISA results	
	Total	Microfilaria (MF)-positive (prevalence in %)	Total	Microfilaria (MF)-positive (prevalence in %)
Ages 5–10	280	3 (1.1)	193	3 (1.6)
Ages 11–20	528	5 (1.0)	386	3 (0.8)
Ages 21–30	491	18 (3.7)	352	11 (3.1)
Ages 31–40	501	25 (5.0)	320	10 (3.1)
Ages 41–50	445	23 (5.2)	293	13 (4.4)
Ages 51–60	287	10 (3.5)	197	4 (2.0)
> 60 years of age	391	9 (2.3)	264	6 (2.3)
Total no. of participants	2,923	93 (3.1)	2,005	50 (2.5)
% female	56 (*n* = 1,646)	48.4 (*n* = 93)	58 (*n* = 1,155)	48 (*n* = 24)
No. of villages	35		35	

### Ov16 ELISA

**Fig. 1:**
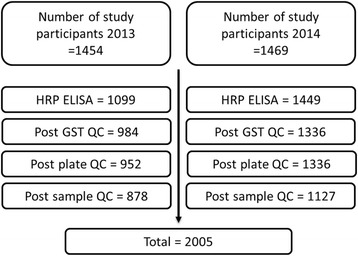
Quality control (QC) selection of specimens used in final analysis for serological response to the Ov16 antigen. Number of study participants are indicated at the top of the figure for the study performed in 2013 and the study performed in 2014 on the left and right, respectively. Progressing top to bottom, the number of specimens used in the ELISA and then remaining after each QC step are indicated

Exposure to onchocerciasis was assessed by HRP Ov16 ELISA for *O. volvulus* antigen Ov16-specific IgG4 in dried blood spots. The recombinant Ov16 is expressed as a GST fusion protein. A GST IgG4 ELISA was also performed on all specimens to screen for the possibility of misclassification due to IgG4 antibody binding to the GST tag on the recombinant Ov16 antigen. HRP Ov16 ELISA and GST ELISA were performed on 1,099 specimens from the 2013 study and 1,449 specimens from the 2014 study. The plates and data set were subjected to a series of quality control steps to form the consolidated dataset (Fig. 1): (i) total number of specimens excluded based on significantly high GST ELISA OD = 228 (9 %); (ii) total number of specimens excluded based on outlier plate control OD = 32 (1 %); and (iii) total number of specimens excluded based on exceeding specimen replicate OD relative difference = 283 (12 %). After all processing, there were ELISA results for a total of 2,005 specimens (Table 1 and Fig. 1).

### Sero-status classification and Ov16 seroprevalence

**Fig. 2:**
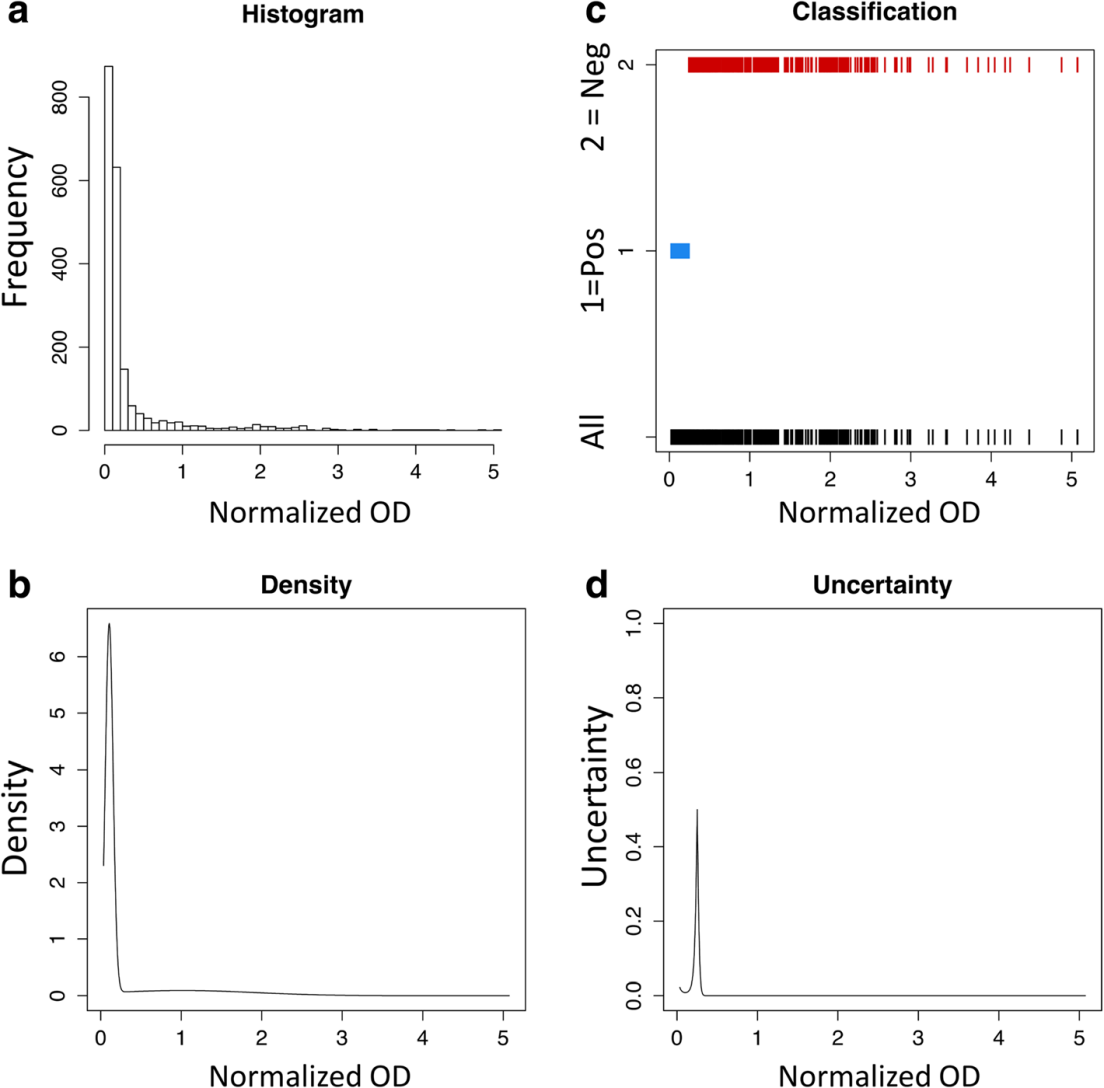
**a** Histogram of normalized absorbance (OD) for horseradish peroxidase (HRP) Ov16 enzyme-linked immunosorbent assay (ELISA) data for participants with Ov16 ELISA results (*n* = 2,005). **b** Density profile for HRP Ov16 ELISA data used for the expectations maximization (EM) mixture model. **c** Classification of specimens for seropositivity resulting from the EM model: 1 represents seropositive, 2 represents seronegative. **d** Uncertainty profile from EM specimens

A dichotomous classification of the combined normalized Ov16 ELISA dataset was performed using an EM mixture model (Fig. 2). The EM method categorized specimens as ELISA-positive (395 samples) or ELISA-negative (1,610 samples), which were then considered to be seropositive or seronegative, respectively. Seropositive prevalence across all age groups was calculated to be 19.7 %.

**Fig. 3:**
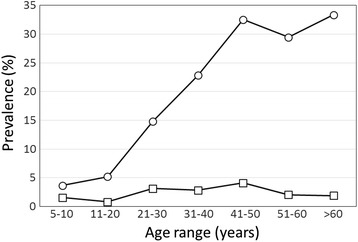
Ov16-specific IgG4 seroprevalence (circles) and microfilaria prevalence (squares) against age groups, for study participants with Ov16 ELISA results (*n* = 2,005)

Overall, the prevalence increased with age, with children under the age of 11 having a seroprevalence of 3.6 % (Fig. 3). One village had only two specimens’ Ov16 ELISA results. Therefore this village was excluded for further analysis and only 2,003 specimens from 34 of the villages were used for further analysis of seroprevalence at individual- and clustered-village level. At the individual village level, the seroprevalence across all age groups ranged from 5 to 54 %. Seroprevalence for children under 11 years of age at an individual village level was not calculated due the low number of children under 11 years recruited in each village.

**Fig. 4:**
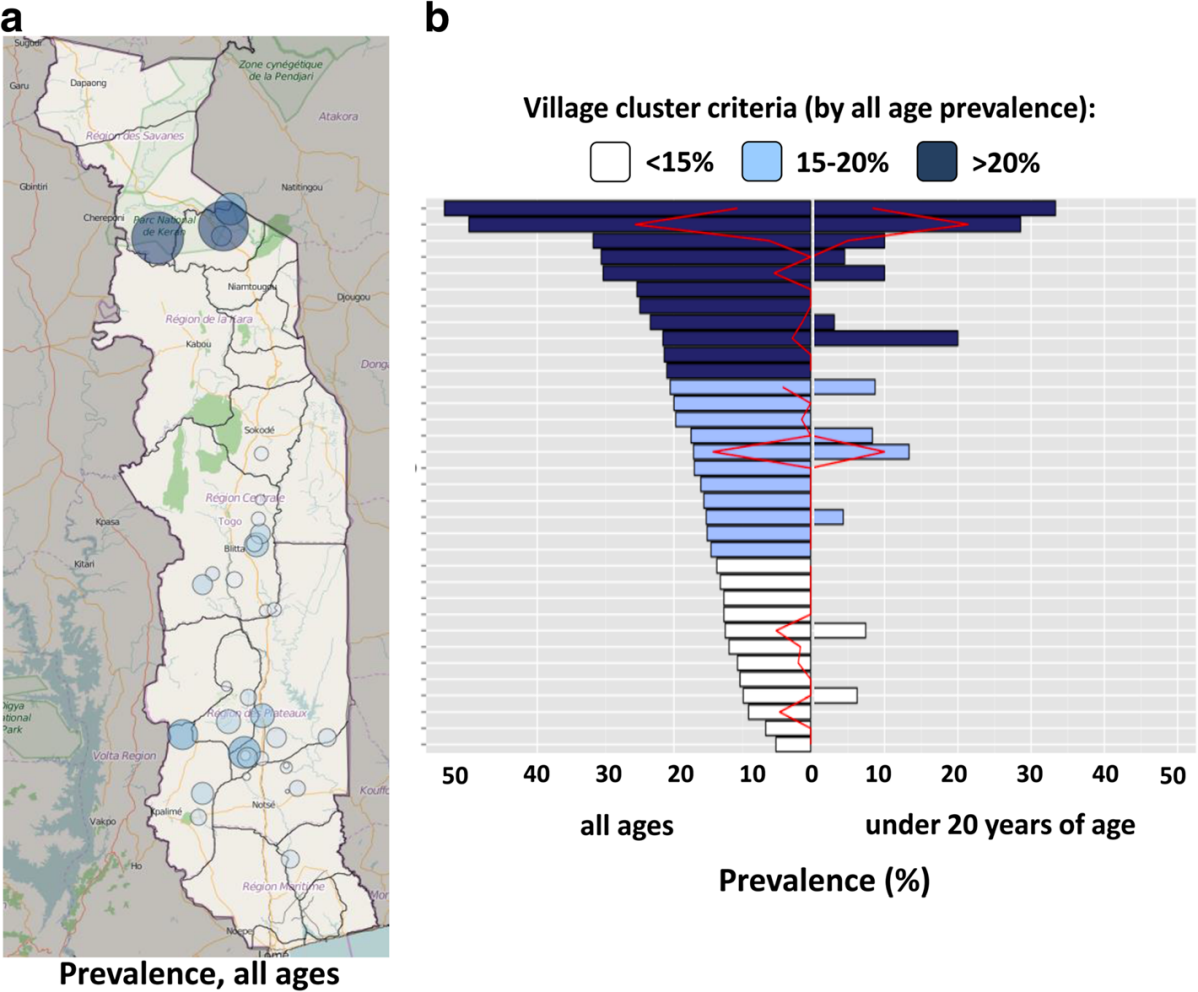
**a** Geographical distribution of villages surveyed; size and color intensity is related to the all-age Ov16 ELISA-positive seroprevalence. **b** Color-coded clustering of villages showing: left of center axis, seroprevalence across all age groups, and right of center axis, seroprevalence for study participants < 20 years of age. The seroprevalence for each age group is aligned side-by-side by village. The microfilaria (MF)-positive prevalences for the villages are shown by the red lines for the respective age groups. Village name and numerical values are provided in Additional file 1: Table S1

To support additional stratification of the results, K-means clustering was used to cluster the villages’ prevalence into three groups; < 15 %, 15–20 % and 21–55 %. The prevalence levels and locations of the villages are shown in Fig. 4. To compare all-age and < 20 years of age groups, seroprevalence and MF-positive prevalence were calculated for the different age group (Fig. 4b, full data set provided in Additional file 1: Table S1). Of the 34 villages included in the analysis, 22 (64 %) no MF-positive specimens were found, and in 29 (85 %) of the villages there was no microfilaridermia observed in the population under 20 years of age.

**Fig. 5:**
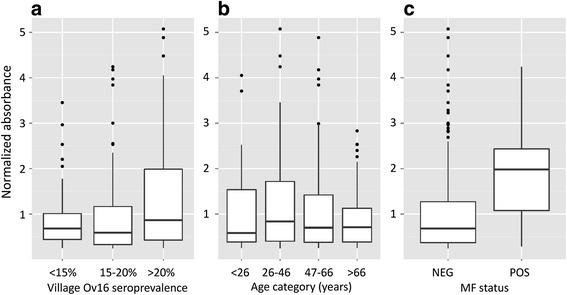
Box plots for normalized anti-Ov16 IgG4 ELISA ODs for different subgroups of ELISA-positive subjects (*n* = 393), as determined by expectation maximization (EM). The solid lines represent median values, the boxes the 25–75 percentiles, and whiskers the minimum to maximum range. Graph **a** shows data for the ≤ 15 %, 15–20 % and > 20 % seroprevalence community clusters; **b** shows data across age ranges (age ranges were selected statistically based on sample distribution); and **c** shows microfilaria (MF)-positive and MF-negative subgroups

Among the anti-Ov16 ELISA positive specimens (*n* = 393), the normalized ODs (defined as specimen OD divided by plate positive control OD) were compared across village prevalence clusters and age groups and between MF positive and MF negative (Fig. 5). Specimens from villages in the cluster with the highest Ov16 antibody prevalence had a higher normalized anti-Ov16 signal overall than the villages with a prevalence of < 15 % (*t* = -3.6754, *df* = 2.5.11, *P* < 0.01), and 15–20 % (*t* = -2.7173, *df* = 208.48, *P* < 0.01). There was no statistical difference in normalized anti-Ov16 signal among the two lower-prevalence clusters. There was no statistical difference in normalized anti-Ov16 signal between age range categories. However, the normalized anti-Ov16 ELISA signal was overall higher in MF-positive specimens than in MF-negative specimens (*t* = -2.7338, *df* = 37.90, *P* < 0.01).

### Seroconversion and seroreversion rate estimations

**Fig. 6:**
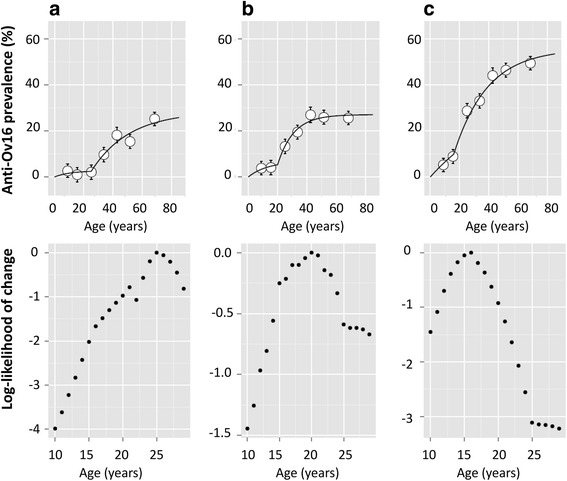
Seroprevalence against age, for the three village clusters described in Fig. 4. Two FOI curves were fitted to each cluster’s seroprevalence plot. Graph **a** shows FOI fits for villages with < 15 % overall seroprevalence; **b** shows FOI fit for villages with 15–20 % seroprevalence; and **c** shows FOI fits for villages with > 20 % seroprevalence. The bars for each point represent the standard deviation. The likelihood of change with respect to age, informing the FOI fits, are shown below the seroprevalence plot for each respective village cluster

Two age-dependent FOI curves were fitted each to the three village prevalence-based clusters identified above. The lower of the two phases of FOI is associated with a younger age group, as reflected by the seroconversion rates for each phase (Table 2). The age at which the optimal transition was found between the two phases of FOI, was highest (older) for the cluster of villages with < 15 % seroprevalence and lowest (younger) for the cluster with the highest all-age seroprevalence, indicating that the state of lower seroconversion occurs in a larger age range for lower-prevalence villages. The bin widths were chosen to keep the bin sizes and numbers equivalent to reduce error of the fitting and allow comparisons between the different cluster FOI models. Fig. 6 shows the best fits for FOI for the seven different age-binned values of Ov16 prevalence for each cluster of villages. Table 2 summarizes the age ranges for the optimal two FOI curve fits and seroconversion rates for the three different clusters shown in Fig. 6.

**Table 2:** Anti-Ov16 IgG4 seroconversion rates resulting from the reverse catalytic model fits for each age group with each village cluster shown in Fig. 6

Village cluster by seroprevalence	1st FOI fit		2nd FOI fit	
	Age range (years)	Seroconversion rate (lambda)	Age range (years)	Seroconversion rate (lambda)
≤ 15 %	5–25	0.0026	> 25	0.01
> 15–20 %	5–20	0.0051	> 20	0.02
> 20 %	5–16	0.0071	> 16	0.02

The age ranges also indicate the optimal point of transition from the first force-of-infection (FOI) fit to the second FOI fit (ages 25, 20, and 16)

### Active infection status by microfilaria status and PCR

PCR was performed on 434 skin snips including 43 skin snips positive for microfilaria by microscopy from a subset of the 2,003 study participants for which there was Ov-16 ELISA data. In this subset, 52 samples were found to be positive by PCR, and PCR and MF microscopy coincided in 30 samples, representing 69.8 % of the 43 skin snip microscopy-positive specimens and 57.7 % of the 52 PCR-positive specimens. Combined, a total of 65 specimens were positive by either PCR or microscopy, such that PCR or skin snip microscopy alone detected 80 % (0.95 CI: 68–88 %) or 66 % (0.95 CI: 53–77 %), respectively, of the combined laboratory-confirmed active infections.

Of the 65 confirmed active infections, 39 (60 %) were positive for Ov16 IgG4 (Table 3). The sensitivity and specificity for Ov16 IgG4 against active infection were 60 % (0.95 CI: 48–71 %) and 75 % (0.95 CI 71–79 %). In this subset, there were 38 specimens from children under the age of 11, five of which were positive either by PCR or MF. For this set of 38 specimens, the sensitivity and specificity of Ov16 IgG4 were 80 % (0.95 CI 28–99 %) and 97 % (0.95 CI 84–100 %), respectively. Both the sensitivity and specificity as a marker for active infection drop as the age range included in their determination is increased (data not shown).

**Table 3:** 2 × 2 contingency tables for anti-Ov16 antibody sensitivity and specificity calculation against microfilarial (MF) status, polymerase chain reaction (PCR) status, and the composite MF + PCR all ages and under 11 year-old

		Ov16 IgG4 all ages		Ov16 IgG4 under age 11	
		Positive	Negative	Positive	Negative
MF	Positive	30	13	3	0
	Negative	100	291	2	33
	Total	130	304	5	33
PCR	Positive	30	22	2	1
	Negative	100	282	3	32
	Total	130	304	5	33
MF + PCR	Positive	39	26	4	1
	Negative	91	278	1	32
	Total	130	304	5	33

## Discussion

In this near-elimination setting, the average total microfilaria counts per MF-positive participant was 12 (range 0–131) and the median was 4, with over 70 % of total microfilaria counts at less than ten counts across both biopsies per participant. More infections were detected by real-time PCR targeting the *O. volvulus* O-150 repeat sequence [18, 19, 40, 41] than by MF by skin snip microscopy, suggesting that PCR is more sensitive to detect active infections, as previously shown [19]. Respective sensitivities for the PCR and skin snip microscopy compared to the composite PCR/MF microscopy positive results, are 80 and 66 %. Some microscopy-positive specimens were not detected by PCR, possibly as a result of using the same residual skin biopsies, which may have lost target DNA or MF through handling. All MF-positives which were missed by PCR had low (< 10) overall combined counts of MF, and the majority had only one or two MF observed, between both biopsies. Our sampling for the PCR analysis was biased, so relative prevalence data are not discussed.

Performance of Ov-16 IgG4 response as a marker for infection was determined using combined PCR-positive and/or MF-positive result as a true positive within the PCR tested samples set. Anti-Ov16 antibody as a marker for active infection, as opposed to (current and previous) exposure to infection, in this population’s study participants that were children < 11 years of age, has a sensitivity and specificity of 80 and 97 %, respectively, although in this age range there were only five true positives. Both sensitivity and specificity of the Ov-16 antibody test drop as a broader age range is included in the analysis - down to 60 and 75 %, respectively, against combined PCR- and microscopy-confirmed active infection for all ages. In older age groups anti-Ov16 antibody response is more reflective of exposure than active infection, explaining why the specificity drops. Anti-Ov16 antibody response was stronger in MF-positive study participants than in MF-negative ones, suggesting that antibody intensity may change temporally following the clearance of MF infection. Longitudinal evidence of the anti-Ov16 IgG4 response is needed to understand age-seroprevalence curves in populations undergoing ivermectin treatment. Furthermore, decreased intensity of the anti-Ov16 response as measured by ELISA may be more likely to be classified as negative. The classification by EM necessarily yields a small group of results which have a higher level of uncertainty surrounding the classification. If a subgroup, of stringent uncertainty greater than 0.05 is filtered from the dataset, removing 151 out of 2,003 results, the performance of the Ov16 ELISA against active infection determined by skin snip microscopy changes from 67.3 % for the unfiltered set to 70.2 % for the filtered set, based on 49 and 47 MF-positive by skin snip microscopy, respectively. The proportion of positive and negative-classified results is similar in the filtered and unfiltered sets. Given the continuous output of ELISA, an ideal analysis method would include an indeterminate results category. However, more data is needed to understand how this indeterminate group may be differentially populated in specific populations’ results or from classification using other methods of data-fitting. Therefore, this study has instead included the intensity of stratified Ov16 ELISA-positive results to compare normalized ELISA signal between categories of interest (Fig. 5).

Overall across all communities and all ages, the MF-positive (as determined by microscopy) prevalence was 2.3 % and Ov16 seroprevalence was 19.7 %. In children under 11, the MF prevalence and the seroprevalence were 1.6 and 3.6 %, respectively, but due to low recruitment of this age group, the confidence intervals around the proportions of positive MF or Ov16 serology are broad. The all-age seroprevalence dynamic range across villages allowed clustering based on village seroprevalence. In all three clusters the seroprevalence increases with age range, as shown previously [28]; however, a distinct two-phase increment was observed. Therefore, fitting seroprevalence against age with two FOI catalytic models provided better fits than a single FOI assumption (data not shown), with the younger age groups within each prevalence cluster experiencing a lower seroconversion rate than the older age groups. A previous FOI model for onchocerciasis included a two-FOI consideration for settings with ongoing transmission as a consequence of epidemiological data suggesting that people under the age of ten years were at lower risk of infection than those older than ten years of age [42]. A dual FOI model applied to trachoma sero-surveillance showed that the age at which the models transitioned coincided with the initiation of MDA activities for trachoma [37]. In the data from this study for surveillance in Togo, the dual seroconversion rates within each age range could be due to either a noticeable decrease in transmission at a particular point in time reflected by the transition age on the fits, or a reflection of a higher exposure risk to infection for the older age groups. These are not mutually exclusive explanations. However, the more constant microfilaria prevalence across all age groups suggests that seroconversion rate changes are indicative of events leading to a change in transmission rates at a village level. Interestingly, the transition age decreases from 25 to 20 to 16 years of age from the low to medium to high prevalence cluster of villages, respectively. Understanding the transition between the two-phases of FOI curves and their relationship to the true transmission in settings approaching elimination but that were highly endemic in the recent past will be important in understanding if there is benefit to extend sero-surveillance to a broader age group. To support this understanding, there is a need to collect all-age seroprevalence data from more settings in Africa.

Clustering the villages by all-age seroprevalence positively coincided with relative seroconversion rates such that the village cluster with the lowest seroprevalence also had the lowest seroconversion rate in the younger age group. While further studies are required to validate the utility of sero-surveillance of older children and adults, this analysis suggests that seroprevalence over a broad age range is useful to stratify relative progression toward elimination across multiple villages within given foci. This may provide a useful means to identify villages that are less responsive for any given reason to current interventions and may require more specific interventions, such as vector control, increased resources for coverage, or twice-a-year MDA. Seroprevalence is increasingly being used to generate models for estimation of transmission dynamics in near-elimination contexts, most notably in malaria [43–46].

## Conclusions

Ov16 antibody response may be a good marker for active infection in children under the age of 11 years in this population. Broadening the age range in seroprevalence studies provides valuable additional information on the progression toward achieving elimination in a given setting. Inclusion of a broader age range increases the number of data points per village and allows better stratification at an individual village level to estimate progression towards elimination based on seroconversion rates. This in turn can inform where and when targeted interventions are required. Similar studies need to be performed in other settings in OCP and APOC countries to develop a better understanding of how to interpret cross-sectional all-age seroprevalence data.

## Abbreviations

DBS, dried blood spots; ELISA, enzyme-linked immunosorbent assay; EM, expectation-maximization; FBS, fetal bovine serum; FOI, force of infection; GST, glutathione-S-transferase; HCl, hydrogen chloride; HRP, horseradish-peroxidase; IQR, interquartile range; LRO, laboratory for onchocerciasis research; MDA, mass drug administration; MF, microfilarial; OD, optical density; Ov, *Onchocerca volvulus*; PBS, phosphate buffered saline; PBST, phosphate buffered saline with Tween; PCR, polymerase chain reaction; RDT, rapid diagnostic test; TMB, 3,3′,5,5’-Tetramethylbenzidine buffer

## Additional file

Additional file 1: Table S1. Village name, number of study participants, Ov16 prevalence and MF prevalence values used to generate Fig. 4b. (DOCX 15 kb)
