# Supplementary material for: Analysis of age-dependent trends in Ov16 IgG4 seroprevalence to onchocerciasis
Source: Parasit Vectors. 2016 Jun 13;9:338. doi: 10.1186/s13071-016-1623-1 (PMC4907250; doi:10.1186/s13071-016-1623-1)
Supplement: Additional file 1: Table S1. — Village name, number of study participants, Ov16 prevalence and MF prevalence values used to generate Fig. 4b. (DOCX 15 kb) [file 13071_2016_1623_MOESM1_ESM.docx]

**Additional file 1: Table S1**. Village name and numerical values for Figure 4B. Villages are listed in the same order from top to bottom as in the figure.

| Village name | All ages | | | Under age 20 | | |
| --- | --- | --- | --- | --- | --- | --- |
|  | **Number of participants** | **Ov16 prevalence**  **(%)** | **MF prevalence**  **(%)** | **Number of participants** | **Ov16 prevalence**  **(%)** | **MF prevalence**  **(%)** |
| Narita | 56 | 54 | 11 | 12 | 33 | 8 |
| Wassite | 74 | 50 | 26 | 14 | 29 | 21 |
| Igbowou Amou | 66 | 32 | 6 | 20 | 10 | 5 |
| Amouta | 62 | 31 | 0 | 22 | 5 | 0 |
| Koffi Ferme | 56 | 30 | 5 | 10 | 10 | 0 |
| Kamalo Cope | 59 | 25 | 0 | 22 | 0 | 0 |
| Kassikide | 76 | 25 | 0 | 13 | 0 | 0 |
| Amougodo | 81 | 23 | 1 | 32 | 3 | 0 |
| Tutu Zionou | 74 | 22 | 3 | 20 | 20 | 0 |
| Kaza | 70 | 21 | 0 | 25 | 0 | 0 |
| Toumouloumon | 38 | 21 | 0 | 19 | 0 | 0 |
| Safou Cope & Atiba | 73 | 21 | 4 | 23 | 9 | 0 |
| Pessiae Ancien | 60 | 20 | 0 | 10 | 0 | 0 |
| Amouto | 76 | 20 | 1 | 11 | 0 | 0 |
| Ananivi Kondji | 40 | 18 | 0 | 12 | 8 | 0 |
| Kpodji | 35 | 17 | 14 | 15 | 13 | 10 |
| Tokpo | 47 | 17 | 0 | 18 | 0 | 0 |
| Kpodji-Cope | 56 | 16 | 0 | 21 | 0 | 0 |
| Aglamassoe | 32 | 16 | 0 | 5 | 0 | 0 |
| Kpendjeria | 72 | 15 | 0 | 23 | 4 | 0 |
| Anamanie | 33 | 15 | 0 | 5 | 0 | 0 |
| Tchanie | 48 | 15 | 0 | 13 | 0 | 0 |
| Kokote | 51 | 14 | 0 | 19 | 0 | 0 |
| Koboyo | 53 | 13 | 0 | 6 | 0 | 0 |
| Game Akeme | 63 | 13 | 0 | 20 | 0 | 0 |
| Kedjebi-Loom | 63 | 13 | 0 | 19 | 0 | 0 |
| Atinkpassa | 80 | 13 | 5 | 27 | 7 | 0 |
| Babame | 67 | 12 | 1 | 20 | 0 | 0 |
| Kouma-Konda | 56 | 11 | 2 | 19 | 0 | 0 |
| Sessaro Kozogade | 58 | 10 | 0 | 9 | 0 | 0 |
| Agnam Cope | 71 | 10 | 0 | 32 | 6 | 0 |
| Kpati Cope | 22 | 9 | 5 | 9 | 0 | 0 |
| Game Togbuihoe | 76 | 7 | 0 | 21 | 0 | 0 |
| Hoevime | 59 | 5 | 0 | 13 | 0 | 0 |
